# Refractory Immune Checkpoint Inhibitor-Induced Colitis Improved by Tacrolimus: A Case Report

**DOI:** 10.3390/healthcare9040418

**Published:** 2021-04-05

**Authors:** Yasuhito Kunogi, Keiichi Tominaga, Keiichiro Abe, Mimari Kanazawa, Takanao Tanaka, Shoko Watanabe, Masayuki Kondo, Akira Kanamori, Makoto Iijima, Kenichi Goda, Yumi Nozawa, Kazuyuki Ishida, Atsushi Irisawa

**Affiliations:** 1 Department of Gastroenterology, Dokkyo Medical University, Tochigi 321-0293, Japan; ykunogi@dokkyomed.ac.jp (Y.K.); abe9841@dokkyomed.ac.jp (K.A.); mimari77@dokkyomed.ac.jp (M.K.); tana1986@dokkyomed.ac.jp (T.T.); shoko-t@dokkyomed.ac.jp (S.W.); m-kondo@dokkyomed.ac.jp (M.K.); k-akira@dokkyomed.ac.jp (A.K.); mkiijima@dokkyomed.ac.jp (M.I.); goda@dokkyomed.ac.jp (K.G.); ishida-k@dokkyomed.ac.jp (K.I.); irisawa@dokkyomed.ac.jp (A.I.); 2 Department of Diagnostics Pathology, Dokkyo Medical University, Tochigi 321-0293, Japan; nozawa-y@dokkyomed.ac.jp

**Keywords:** immune checkpoint inhibitor-induced enterocolitis, lung cancer, pembrolizumab, tacrolimus

## Abstract

Immune checkpoint inhibitors (ICIs) increase T-cell activity and antitumor immune response. However, they also have immune-related adverse effects that can affect the gastrointestinal (GI) tract. A 62-year-old male patient who had undergone right lung upper lobectomy for adenocarcinoma of the lung received chemotherapy with pemetrexed sodium hydrate, carboplatin, and pembrolizumab to prevent postoperative recurrence of liver metastasis. However, the patient experienced severe diarrhea four months after the start of chemotherapy. Although a corticosteroid and two biological preparations were administered to alleviate the diarrhea, no improvement was observed. Eventually, remission was achieved when tacrolimus was administered. Treatment with corticosteroids is recommended for patients with GI adverse effects of ICIs. Rapid introduction of infliximab is necessary for refractory patients. Nevertheless, for refractory cases such as that of our patient, for whom even this regimen is inefficacious, tacrolimus might be recommended to induce remission as with cases of ulcerative colitis.

## 1. Introduction

Since the introduction of immune checkpoint inhibitors (ICIs) such as nivolumab and pembrolizumab as treatment for carcinomas of various types including malignant melanoma and non-small cell lung cancer, reports have described widely various immune-related adverse events (irAEs). These drugs increase T-cell activity and antitumor immune response. However, they also have immune-related adverse effects that can affect the gastrointestinal (GI) tract. Collins et al. [1] reported in a systematic review that these adverse effects were observed in 7–30% of patients treated with ICIs. Abdel-Rahman et al. [2] performed a meta-analysis and demonstrated that ICIs are associated with significantly increased risk of all-grade and high-grade colitis. Treatment of patients with GI adverse effects of ICIs is recommended with corticosteroids, and with rapid introduction of infliximab therapy for non-responders. This report describes a case of refractory ICI-induced colitis (pembrolizumab (PEMB)-induced colitis) that was resistant to corticosteroid and two biological preparations, following which remission was achieved with the administration of tacrolimus. Although several reports have described that the use of tacrolimus can induce remission [3], few have been presented. We herein report a case in which tacrolimus administration contributed to improvement of refractory ICI-induced colitis.

## 2. Case Report

A 62-year-old man who had undergone right lung upper lobectomy for adenocarcinoma of the lung (Stage Ib, pT2aN0M0) received chemotherapy comprising pemetrexed sodium hydrate 930 mg, carboplatin 700 mg, and PEMB 200 mg. Each was administered by infusion at four-week intervals to prevent postoperative recurrence of liver metastasis. Following the fourth chemotherapy session, the patient experienced diarrhea. He was hospitalized after oral antidiarrheal medication failed to elicit in any improvement. The patient had a smoking history from age 20 to age 57 years (30 cigarettes/day). The blood test upon hospitalization (Table 1) revealed a decreased state of nutrition, with total protein and albumin falling respectively to 6.3 g/dL and 3.2 g/dL.

**Figure 1:**
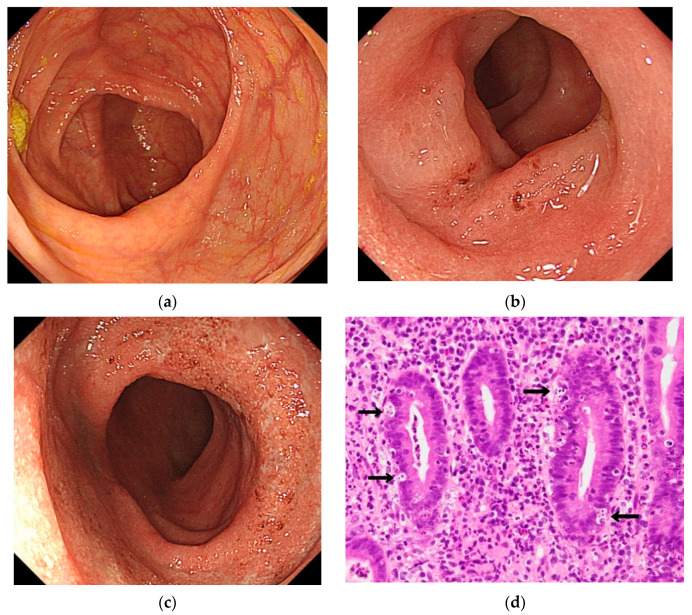
First colonoscopy findings after admission. Continuous coarse granular mucosa up to the sigmoid colon is observed. Apoptosis (arrows) is readily apparent in the pathological image. Pathological findings include epithelial apoptosis, infiltration of mixed inflammatory cells consisting of neutrophils, lymphocytes, and plasma cells, cryptitis, and crypt abscess. The apoptotic image shows the tendency of miniaturization and fusion of the crypt epithelium. The apoptoses was 14 per 10 crypts: (**a**), cecum; (**b**), sigmoid colon; (**c**), rectum; (**d**), pathological image of sigmoid colon.

Although no renal dysfunction was noted with a urea nitrogen level of 7.1 mg/dL and a creatinine level of 0.74 mg/dL, the C-reactive protein level was high at 4.36 mg/dL, indicating an inflammatory reaction. Additionally, the patient’s white blood cell count was 2.6 × 10^9^/L. The hemoglobin concentration was 8.9 g/dL. These low concentrations were indicative of chemotherapy effects. Colonoscopy (CS) performed on Day Five of hospitalization revealed continued edema from the sigmoid colon to the rectum and continued inflammation accompanied by redness (Figure 1a–c). In biopsied tissue, there were an ulcerative colitis-like crypt abscess, cryptitis, and branching, flexure, and deformity of glandular ducts with highly developed neutrophils, plasmacytes, and lymphocytic infiltration. There were also findings indicative of high levels of apoptosis on the basal side of the epithelium. Overall, these findings were consistent with the diagnosis of ICI-induced colitis (Figure 1d).

**Figure 2:**
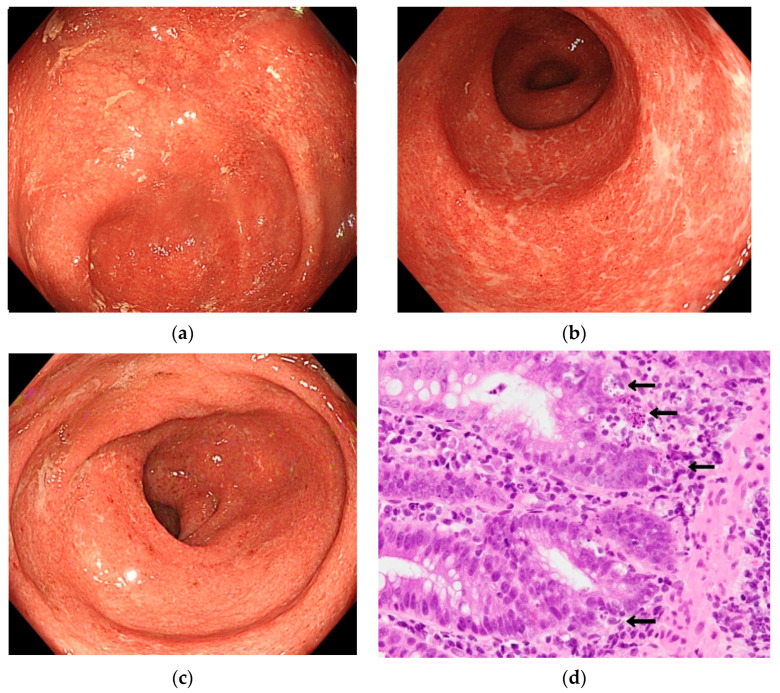
Endoscopic findings when the symptoms were most severe. The fine granular mucosa extends continuously from the rectum to the cecum. A hexagonal ulcer formation is also observed in part of the sigmoid colon. The degree of apoptosis (arrows) was the same as that of the prior colonoscopy. The apoptoses were 10 per 10 crypts: (**a**) cecum; (**b**) sigmoid colon; (**c**) rectum; (**d**) pathological image of sigmoid colon.

Because causes other than ICIs were inconceivable to explain the colitis, the patient was diagnosed with ICI-induced colitis. Despite the administration of 5-aminosalicylic acid (5-ASA) 4800 mg/day by oral administration in the morning, no improvement was observed. On day 20 of hospitalization, prednisolone (PSL) was initiated at 70 mg/day by intravenous administration in the morning. Nevertheless, the introduction of PSL did not engender any improvement in the condition. Diarrhea occurred at least 10 times for consecutive days, with subsequent deterioration of the nutritional status. Therefore, it was inferred that the condition was PSL-resistant. For that reason, PSL was tapered off. Next, infliximab (IFX) was administered (400 mg) on day 30 by intravenous administration in 60 min. Although IFX was readministered two weeks later, no improvement was observed. CS performed on Day 49 revealed highly developed inflammation covering the entire large intestine (Figure 2a–c). Pathologically, no change was found in the degree of inflammation. (Figure 2d).

**Figure 3:**
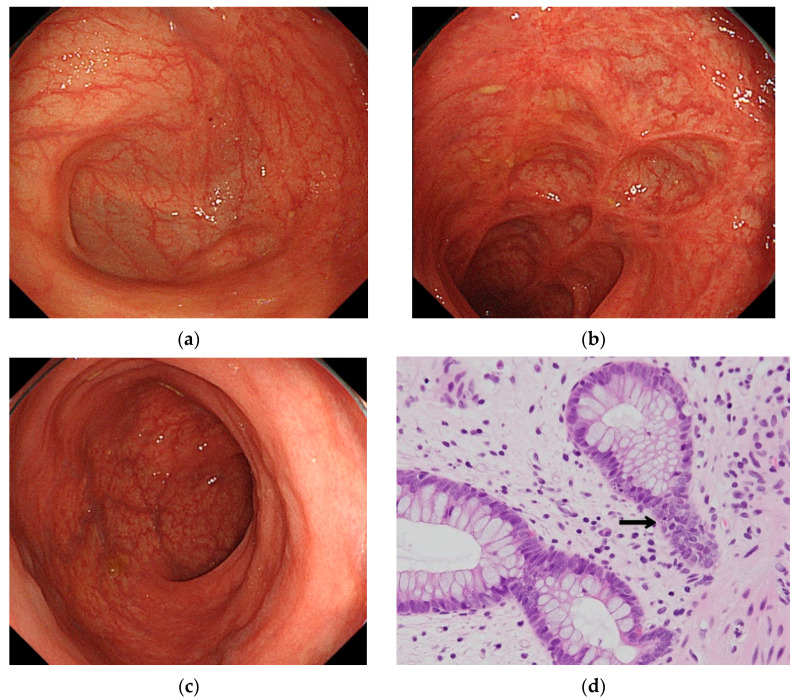
Colonoscopic findings after remission. No inflammatory finding was observed in the entire colon, but scarring is observed in areas with high inflammation, such as the cecum and sigmoid colon. The histopathological image depicts a few inflammatory cells without neutrophils. The apoptosis was decreased (arrow). The apoptoses were 1 per 10 crypts: (**a**) cecum; (**b**) sigmoid colon; (**c**) rectum; (**d**) pathological image of sigmoid colon.

**Figure 4:**
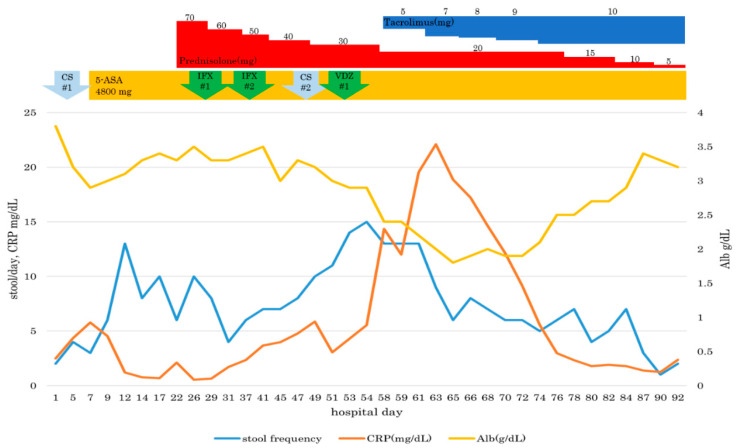
Clinical course until discharge.

On Day 53, we administered a second biological preparation, vedolizumab (VDZ) (300 mg) by infusion in 30 min. However, no improvement of the patient’s diarrhea was observed. Later, oral administration in the morning of tacrolimus, an immunosuppressant, was initiated (6 mg/day) from Day 58. We continued to administer adjusted doses of tacrolimus aimed at achieving a blood concentration of 10–15 ng/mL. Consequently, diarrhea improved gradually. The stools were well-formed, with frequency stabilizing to 1–2 times per day. Blood tests detected an improved albumin level of 3.2 g/dL. On day 92, the patient was discharged from the hospital in a good general condition. After discharge, appropriate blood concentrations of tacrolimus were maintained at a dosage of 7 to 8 mg/day. No adverse event attributable to tacrolimus was observed after administration. The colitis symptoms also seemed to improve. Following remission, CS performed 42 days after discharge revealed that the fine granular membrane had disappeared throughout the entire colon. In the ascending and descending colon, highly developed cicatricial changes were observed as the inflammation resolved. As an evaluation of pathological inflammation, a well-experienced pathologist counted the apoptotic images every 10 crypts. The first pathological specimen showed up to 14 apoptosis. The second pathological specimen also showed up to 10 apoptosis. However, only one incidence of apoptosis was observed in the pathological specimen after administration of tacrolimus. Results showed that the inflammatory cells infiltrated slightly. Therefore, these findings suggest that, from a pathological perspective, the treatment had been successful (Figure 3a,b). Figure 4 shows the clinical course of our patient.

## 3. Discussion

Recently, several ICIs, including anti-cytotoxic T lymphocyte antigen 4 (CTLA-4) antibody, anti-programmed cell death protein-1 (PD-1) antibody, and anti-PD ligand-1 (PD-L1) antibody, have become available for clinical use. PEMB used in this case is an anti-PD-1 antibody that became available following nivolumab. Its excellent antitumor effects have been demonstrated by two Phase-III clinical studies targeting non-small cell lung cancer [4,5].

Three recent systematic literature reviews and meta-analyses of published studies (including controlled trials) have assessed the risks of diarrhea and colitis in patients treated with anti-PD-1 and/or anti-CTLA-4. The incidence of diarrhea was 12.1–13.7% for anti-PD-1 and 30.2–35.4% for anti-CTLA-4. The incidence of colitis was 0.7–1.6% for anti-PD-1, 5.7–9.1% for anti-CTLA-4%, and 13.6% for the combination of both therapies [6,7,8]. Because the number of diseases treated with ICIs increases, gastroenterologists will see more patients with ICI-induced GI adverse events. These drugs enhance antitumor T-cell activity and therefore induce irAE, of which GI irAE are among the most frequent and severe. Eigentler et al. [9] reported the median duration until onset of colitis after the use of an anti-PD-1 antibody as 18.1 weeks. According to a report summarizing 131 cases that underwent monotherapy with ipilimumab for malignant melanoma, 34.4% of the patients had developed irAEs (Grades 2 to 5) at 70 days or longer after the final administration of ipilimumab [10]. Anti-PD-1 and anti-PD-L1 antibodies are believed to exert their antitumor effects by blocking such PD-1/PD-L1 signaling and thereby releasing the suppression of effector T cells [11,12]. Moreover, anti-PD-a antibodies activate naïve T cells. Although this action maintains antitumor effects even after discontinuation of anti-PD-1 antibodies, it has also been reported as related to the persistence and delayed onset of adverse reactions [13]. Our patient developed colitis accompanied by high frequency of diarrhea approximately four months after the initial administration of PEMB. It remains unclear whether the passage of time or other causes were responsible. Therefore, the condition was diagnosed as ICI-induced colitis.

The frequency of ICI-related gastrointestinal disturbance is regarded as different depending on the mechanism of the drug used. GI irAE induced by anti-CTLA-4 are frequent, potentially severe (8–22%), and similar to irritable bowel disease, whereas those induced by PD-1 blockade are apparently less frequent and clinically more diverse [14,15]. Moreover, a meta-analysis of articles reporting the use of PD-1/PD-L1 inhibitors against multiple malignant tumors including lung cancer revealed that the incidence of PEMB-induced diarrhea was lowest, at approximately 4%. The incidence of severe colitis was below 1% [16]. However, Boutros et al. [17] reported that, unlike cases of ICI-induced colitis by anti-CTLA-4 ipilimumab and anti-PD-1 nivolumab, all cases by anti-PD-1 PEMB were severe cases of colitis (Grade 3 or above). Although the incidence of colitis caused by PEMB might be lower than that of ipilimumab, once PEMB-induced colitis develops, it might present a more serious prognosis than ICI-induced colitis by other drugs. In the case described herein, administration of 5-ASA preparations, steroids, and biological preparations yielded no sign of improvement; consequently, we faced difficulty in treating this case, which had a severe prognosis. Although it constitutes only mere speculation, this refractory nature might be one characteristic of ICI-induced colitis by PEBM.

The characteristic endoscopic findings of ICI-induced colitis include the disappearance of blood vessel transparency and a granular membrane resembling ulcerative colitis. Pathological findings include diffuse or patchy infiltrates of neutrophils, crypt abscess, cryptitis, and apoptosis [7,8,18]. Yanai et al. reported apoptosis as an important histological finding of ICI-induced colitis [18]. In this case, highly developed apoptosis was observed on the basal side of the epithelium, thereby exhibiting a pathological trait of ICI-induced colitis.

This case exhibited therapeutic resistance to PSL, IFX, and VDZ. The symptoms eventually improved with the administration of tacrolimus. No clinical practice guidelines have been established for the treatment of ICI-induced diarrhea/colitis. Normally, the first course of action in a suspected case of ICI-induced colitis is to discontinue ICI, which is the causative drug. If the symptoms persist or worsen, then the use of 5-ASA and PSL is required. If the treatment described above achieves no improvement, then administration of biological preparations should also be considered. Several reports of studies have described the efficacy of IFX and VDZ against ICI-induced colitis [19,20,21,22]. According to earlier reports, many cases of ICI-induced colitis were improved by the treatment described above [15]. However, when IFX and VDZ prove ineffective, as in this case, the next course of action remains unclear for the most part. Regarding the effectiveness of immunosuppressants (e.g., tacrolimus used for this study) against irAEs, there have been reports of remission reached by tacrolimus against skin toxicity [23] or improvement in PSL/IFX-resistant nivolumab-induced colitis by oral administration of cyclosporine [24]. For the patient described herein, refractory ICI-related colitis was improved using tacrolimus. Although tacrolimus is generally regarded as a medication for moderate-to-severe cases of steroid-resistant ulcerative colitis [25], the British Society of Gastroenterology and European Society for Medical Oncology recently recommended tacrolimus as a therapeutic agent for refractory ICI-induced Colitis [26,27]. The use of tacrolimus in this case was regarded as clinically appropriate. We administered two biological agents to our patient following PSL, but no improvement in inflammation was observed. In contrast, rapid aggravation of the symptoms and inflammatory reaction required urgent changes in the therapeutic regimen. Because the endoscopic and clinical images resembled those of ulcerative colitis, we developed a treatment strategy based on guidelines for ulcerative colitis. Consequently, tacrolimus was selected. Tacrolimus forms an intracellular complex with FK506 binding protein 12 and binds to calcineurin, thereby inhibiting the intracellular transfer of nuclear factors of activated T cells and suppressing the production of cytokines such as IL-2, TNF-α, and IFN-γ [28,29,30]. Consequently, this drug exerts immunosuppressive effects by inhibiting the differentiation and proliferation of T cells. In this study, however, T cells were activated by PEMB. They became damaging to tissues. It was likely that the clinical symptoms improved after tacrolimus suppressed those effects. Although remission was maintained for three months after the start of tacrolimus, liver metastasis is increasing. Depending on the patient’s general condition, restarting chemotherapy might be an option. However, no report has described recurrence of ICI-induced colitis under tacrolimus administration, the patient’s response must be observed carefully. In addition, tacrolimus is known to have several adverse effects such as neurotoxicity, hypertension, tremor, and nausea [28].

Our patient exhibited refractory colitis induced by PEMB, which was resistant to internal medical treatment, specifically comprising biological preparations. Ultimately, symptoms improved following tacrolimus administration. Several reports have described tacrolimus use for ICI-induced colitis [3,31]. We summarized the reported cases of ICI-induced colitis for which tacrolimus was administered (Table 2).

Although the incidence of colitis because of PEMB is rare, cases tend to become more severe than those involving ICI-induced colitis of other types, making early therapeutic interventions fundamentally important. When a refractory case is noted during the early stages of treatment, the administration of tacrolimus should be considered, as it was for our patient.

## Figures and Tables

**Table 1:** Laboratory test data on admission.

AST	28 U/L	Na	132 mmol/L
ALT	18 U/L	K	4.1 mmol/L
LDH	279 U/L	Cl	95 mmol/L
UN	7.1 mg/dL		
Cre	0.74 mg/dL	WBC	2.6 × 10^9^/L
TP	6.3 mg/dL	RBC	2.80 × 10^12^/L
Alb	3.2 mg/dL	Hb	8.9 g/dL
CRP	4.36 mg/dL	Plt	33.8 × 10^4^/L

Abbreviations: AST, aspartate aminotransferase; ALT, alanine aminotransferase; LDH, lactate dehydrogenase; UN, urea nitrogen; Cre, creatinine; TP, total protein; Alb, albumin; CRP, C-reactive protein; Na, sodium; K, potassium; Cl, chloride; WBC, white blood cell; RBC, red blood cell; Hb, hemoglobin; Plt, platelet.

**Table 2:** Literature review of gastrointestinal immune-related adverse events for which tacrolimus was administered.

Case	Year	Author	Causative Drug	Symptom	Treatment	Outcome
#1	2016	Beardslee et al. [3]	ipilimumab and nivolumab	colitis	Steroids, IFX and Tac	Colitis improved.
#2	2016	Beardslee et al. [3]	nivolumab	colitis	PSL, IFX, and Tac	Colitis improved.
#3	2017	Beardslee et al. [3]	ipilimumab and nivolumab	oral mucositis	PSL, IFX, and Tac	Oral mucositis improved.
#4	2018	Sano et al. [31]	pembrolizumab	colitis	PSL, IFX, GMA, and Tac	Colitis did not improve.
#5		our case	pembrolizumab	colitis	5-ASA, PSL, IFX, VDZ, and Tac	Colitis improved.

Abbreviations: 5-ASA, 5-aminoaslicylic acid; GMA, granulocyte and monocyte adsorptive apheresis; IFX, infliximab; PSL, prednisolone; Tac, tacrolimus; VDZ, vedolizumab.

## Data Availability

Not applicable.
